# Psychological Stress on Female Mice Diminishes the Developmental Potential of Oocytes: A Study Using the Predatory Stress Model

**DOI:** 10.1371/journal.pone.0048083

**Published:** 2012-10-31

**Authors:** Yu-Xiang Liu, Ya-Nan Cheng, Yi-Long Miao, De-Li Wei, Li-Hua Zhao, Ming-Jiu Luo, Jing-He Tan

**Affiliations:** College of Animal Science and Veterinary Medicine, Shandong Agricultural University, Tai-an City, People's Republic of China; VU University Medical Center, The Netherlands

## Abstract

Although the predatory stress experimental protocol is considered more psychological than the restraint protocol, it has rarely been used to study the effect of psychological stress on reproduction. Few studies exist on the direct effect of psychological stress to a female on developmental competence of her oocytes, and the direct effect of predatory maternal stress on oocytes has not been reported. In this study, a predatory stress system was first established for mice with cats as predators. Beginning 24 h after injection of equine chorionic gonadotropin, female mice were subjected to predatory stress for 24 h. Evaluation of mouse responses showed that the predatory stress system that we established increased anxiety-like behaviors and plasma cortisol concentrations significantly and continuously while not affecting food and water intake of the mice. In vitro experiments showed that whereas oocyte maturation and Sr^2+^ activation or fertilization were unaffected by maternal predatory stress, rate of blastocyst formation and number of cells per blastocyst decreased significantly in stressed mice compared to non-stressed controls. In vivo embryo development indicated that both the number of blastocysts recovered per donor mouse and the average number of young per recipient after embryo transfer of blastocysts with similar cell counts were significantly lower in stressed than in unstressed donor mice. It is concluded that the predatory stress system we established was both effective and durative to induce mouse stress responses. Furthermore, predatory stress applied during the oocyte pre-maturation stage significantly impaired oocyte developmental potential while exerting no measurable impact on nuclear maturation, suggesting that cytoplasmic maturation of mouse oocytes was more vulnerable to maternal stress than nuclear maturation.

## Introduction

Studies suggest that psychological stress can exert detrimental effects on reproduction in women. For example, thin women with a poor psychosocial profiles are at increased risk of giving birth to low birth weight and preterm infants when depressed during pregnancy [1], and psychosocial stress during pregnancy is associated with spontaneous preterm birth and low birth weight even after adjustment for maternal demographic and behavioral characteristics [2]. Psychological stress among women undergoing in vitro fertilization (IVF) or gamete intra-fallopian transfer [3] has often been associated with a decrease in number of oocytes retrieved and fertilized, as well as in pregnancy rate, live birth delivery and birth weight [4]. Furthermore in comparison to fertile controls, infertile women have been reported to have a higher incidence of personality profiles including greater suspicion, guilt and hostility and higher levels of circulating prolactin and cortisol [5], [6].

Restraint of animals as an experimental procedure is known to induce psychogenic stress [7], [8]. For example, mice exposed to 5 h of restraint on days 1–3, 4–6, or 1–6 of pregnancy showed increased proportions of abnormal corpora lutea, decreased serum progesterone concentrations, reduced pregnancy rates and lower litter size [9]. In rats, restraint stress during mid-pregnancy caused luteolysis, retarded fetal development and even fetal loss [10], [11]. Regrouping group-housed sows after weaning created stressful situations and elevated blood cortisol concentration [12], and in such sows rebreeding was performed more often after an irregular estrus-to-estrus interval than in sows kept in individual stalls [13]. In sheep, psychological stress applied to ewes for 1 h on days 2 and 3 after conception affected fetal growth and gestation length [14].

According to Hobel and Culhane [15], stressors present early in pregnancy were often present before conception as well. Hence, to improve reproductive outcomes, stress preventing or reducing interventions should begin before conception. In fact, a prospective study indicates that stressful life events in women may reduce the chances of a successful outcome following IVF, possibly through psychobiological mechanisms affecting medical end-points such as oocyte retrieval outcome [16]. However, reports concerning the direct effect of psychological stress on oocyte development and maturation are few, and all studies reported so far were conducted inducing stress through restraint [17]–[19]. In addition, although studies indicate that stress can alter cortisol excretion patterns during the estrous cycle which ultimately affects the hormonal profile in critical stages of the reproductive process [20]–[22], the mechanisms by which stress impairs oocyte competence are largely unknown. Furthermore, although restraint is widely used as a psychological stressor, it is often implicated as a cause of physiological insults. It would therefore be beneficial to use a stress system that is primarily psychological to study the effect of psychological stress on oocyte developmental potential.

Unlike the restraint procedure, the predatory stress system induces a pure psychological stress without directly inducing any physiological stress. In a typical predatory stress procedure, for example, freely moving animals are placed in a cage within a larger cage containing a predator. In this situation, prey animals could not be physically harmed but would experience fear. Predator stimuli are clearly stressful for rodents. As an example, exposure of rats and mice to natural predators or to their odors induces anxiety-like states [23]–[27], and rats avoid cat odor sources [28] and display high rates of risk assessment, including flat back approach and stretch-attend behaviors oriented toward the threatening odor [29]. Exposure of rats to predator odors also results in anxiogenic responses when subjects are tested in the absence of odor shortly afterward in social interaction and in elevated plus-maze (EPM) tests [30]. Chronic exposure to rat odor in mice also induces anxiogenic responses in the EPM [31]. Furthermore, exposure to natural predators and their odors induces a pattern of monaminergic and stress hormonal elevations in both rats [32] and mice [33], [34]. However, the effect of psychological stress on oocyte development and maturation has not been studied using a predatory stress procedure.

In most mammals, oocytes enter the early stages of meiosis during fetal life and subsequently become arrested at the dictyate stage of prophase I until birth, after which some of them begin to grow [35]. The fully-grown oocyte contains a single large nucleus or germinal vesicle (GV), and it will resume meiosis following exposure of follicles to gonadotropin stimulation. Resumption of meiosis results in GV breakdown (GVBD) followed by formation of the first polar body and the mature metaphase II oocyte. Studies in cattle indicate that oocyte development in dominant follicles immediately before the LH surge (the pre-maturation stage) is crucial for oocytes to acquire developmental competence [36]. Furthermore, our recent study demonstrated that after female mice were injected with eCG, oocytes were more vulnerable during the second 24-h period than during the first 24-h period to the restraint-induced impairment of oocyte competence [19]. In the current study, a predatory stress system was first established for mice with cats as predators. This system and its performance were then evaluated by the EPM test and measurements of serum cortisol. Effects of predatory stress applied during the second 24-h period following eCG administration on oocyte maturation, activation/fertilization and embryo development in vitro and in vivo were recorded. The results indicated that the predatory stress system we established evoked dependable stress responses in mice and that maternal predatory psychological stress significantly diminished oocyte developmental potential.

## Results

### Exposure to a Cat Increased Anxiety-like Behaviors in Mice

**Figure 1:**
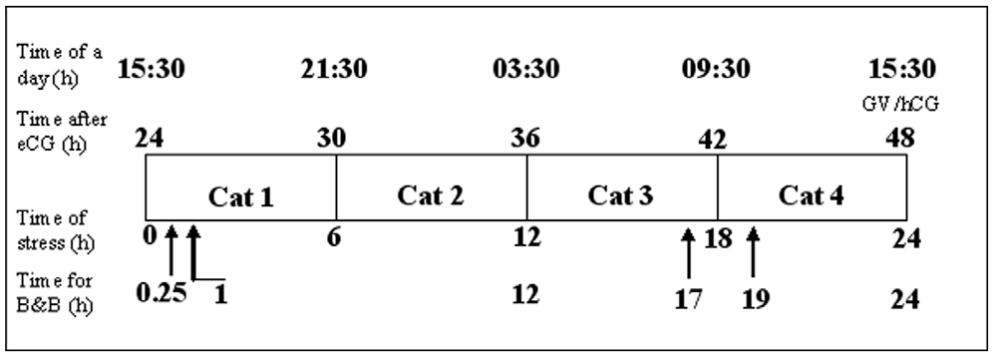
A timetable for different experimental procedures relative to the animals’ light-dark cycle. Female mice were injected with eCG at 15∶30. Mice in the experimental group were subjected to predatory stress for 24 h from 24 h (15∶30 the next day) to 48 h after eCG injection. To ensure continuity of stress, the predator cat was replaced every 6 h during the 24-h stress period with an individual to which the mice had not been exposed. Blood collection and 5-min behavior tests (B&B) were conducted at 0.25 (15∶45), 1 (16∶30), 12 (03∶30), 17 (08∶30), 19 (09∶30) and 24 (15∶30) h of stress. At 48 h after eCG injection, oocytes at the germinal vesicle (GV) stage were collected for maturation in vitro, and some females were injected with hCG and caged with males to produce oocytes for subsequent evaluation of development in vivo.

To evaluate the stress response of mice to our predatory stress system, mouse anxiety-like behaviors were examined by EPM tests. Female mice were exposed to a cat starting 24 h following eCG injection (Fig. 1), and their EPM performance was recorded for 5 min at specific times after initial cat exposure. The level of anxiety was estimated by both the number of entries and the time spent in the open arm of the maze [37], [38]. Results showed that both the number of entries and the time spent in the open arm of the maze decreased significantly after exposure to a cat at each of the times examined (Table 1), suggesting predatory stress increased anxiety-like behaviors of mice. It should be noted that the number of entries and time spent in the open arm were not restored after 1-h recovery from the 24 h exposure to the cats (24+1 h).

**Table 1:** Elevated plus maze (EPM) results after mice were exposed to predators for different times starting 24 h following eCG injection.

Time post-initiation of stress	Treatment	Mice examined	Open arm		Closed arm	
			Time (s)	Entries	Time (s)	Entries
15 min	Control	15	53.9±5.4^a^	20.6±2.5^a^	157.5±14.3^a^	24.2±4.6^a^
	Stressed	16	7.4±1.8^b^	3.7±1.0^b^	267.6±5.5^b^	11.1±2.0^b^
17 h	Control	18	48.8±5.6^a^	21.2±2.8^a^	155.9±11.6^a^	30.2±2.9^a^
	Stressed	20	15.7±3.2^b^	10.7±2.2^b^	219.4±9.1^b^	21.0±3.1^b^
19 h	Control	17	63.2±4.3^a^	24.0±3.0^a^	143.9±13.5^a^	24.9±3.5^a^
	Stressed	21	17.1±2.7^b^	9.2±1.4^b^	243.2±6.9^b^	15.0±2.5^b^
24 h	Control	13	58.6±4.8^a^	16.2±2.0^a^	157.9±8.8^a^	24.8±1.7^a^
	Stressed	14	22.5±5.7^b^	8.8±1.4^b^	216.4±10.1^b^	21.5±2.8^a^
24+1 h	Control	14	48.6±6.5^a^	15.4±1.3^a^	163.6±7.0^a^	23.9±2.4^a^
	Stressed	15	15.1±3.6^b^	7.1±1.2^b^	209.2±8.3^b^	22.6±2.7^a^

a–c: Values without a common letter in their superscripts differ significantly (P<0.05) within columns.

### Exposure to the Cats Increased Plasma Cortisol Concentrations of Mice

**Figure 2:**
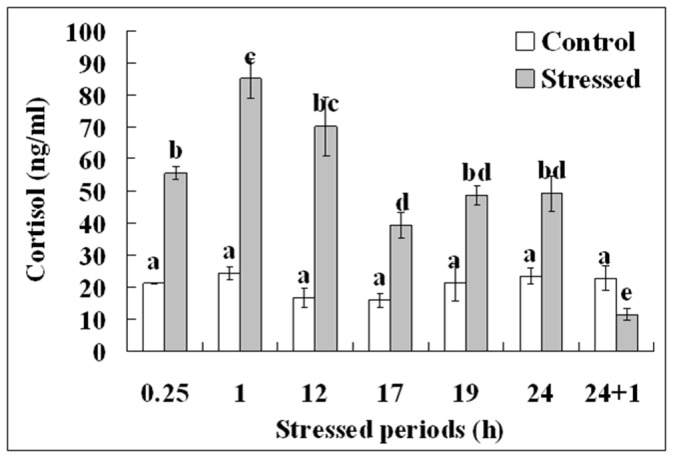
Serum cortisol concentrations after mice were exposed to cats for different times starting from 24 h following eCG injection. Each treatment was repeated 6 times using blood samples from 6 individual mice. Values without a common letter above their bars differ significantly (P<0.05).

To further confirm the effectiveness of our predatory stress system, plasma cortisol concentrations were measured after mice had been exposed to the cat stress. Female mice were exposed to cats starting 24 h after eCG injection (Fig. 1), and their plasma cortisol was measured by radioimmunoassay at various times after the onset of cat exposure. Whereas the cortisol level in control mice did not change significantly across the 24 h period, cortisol concentrations in stressed mice increased significantly by 15 min and peaked at 1 h and 12 h after initial cat exposure (Fig. 2). Cortisol concentration in stressed mice declined thereafter but remained significantly higher than that of their unstressed counterparts up to 24 h of cat exposure. The cortisol concentrations in control mice were relatively lower at 12 h and 17 h than at other times during the experiment. This might be due to the fact that blood samples were collected early in the morning when mice were stressed for 12 h (03∶30) and 17 h (08∶30). Serum cortisol in stressed mice decreased to below the level of control mice after a 1-h recovery following cat exposure for 24 h (24+1 h). Together, the results suggested that our predatory stress system evoked an ongoing stress response in mice during the 24-h period of cat exposure.

### Our Predatory Stress System did not Affect Food and Water Intake of Mice

To test whether our predatory stress system would affect feeding and whether a food and water deprivation control would be necessary for future experiments, the stressed (n = 22) and control (n = 22) mice were kept for 24 h starting from 24 h after eCG injection in special prey cages with the floor covered by a pressboard. Food (including that crushed on the floor) and water were weighed both before and after experiments. The results indicated that the average intake of food (4.6±0.3 vs. 5.5±0.3 g) and water (5.9±0.3 vs. 6.3±0.3 ml) did not differ significantly (P>0.05) between stressed and the control mice, respectively.

### Effects of Maternal Predatory Stress During Follicular Growth and Maturation on Oocyte Maturation and Embryo Development *in vitro*

To study the effects of maternal predatory stress during follicular growth and maturation on oocyte maturation and embryo development in vitro, mice were exposed to the stress for 24 h, starting 24 h after eCG administration (Fig. 1). Oocytes at the GV stage were collected for in vitro maturation and fertilization/activation immediately at the end of the stress period. Oocyte maturation rates were high and did not differ between control (97.4±1.4, 115/118) and stressed mice (96.8±0.9, 118/121). Whereas activation/fertilization rates were not affected by predatory stress, rate of blastocyst formation and cell counts per blastocyst following either Sr^2+^ activation or in vitro fertilization were significantly lower in stressed mice compared to the non-stressed controls (Table 2). The results indicate that although predatory stress applied to mice at the pre-maturation stage of oocyte development had no effect on oocyte nuclear maturation, stress did significantly impair oocyte cytoplasmic maturation.

**Table 2:** Oocyte activation or fertilization and embryo development in vitro after mice were exposed to cat stress for 24 h starting from 24 h following eCG injection.

Oocyte treatment	Mouse treatment	Oocytes cultured	% Oocytes activated/fertilized (n)	% Blastocysts (n)	Cell number/blastocyst
Activation	Control	115	98.1±1.3 (113)^a^	26.7±0.3 (30)^a^	38.2±2.9^a^
	Stressed	118	97.1±1.5 (116)^a^	8.1±1.7 (11)^b^	23.6±1.7^b^
Fertilization	Control	136	92.6±2.3 (127)^a^	87.1±2.4 (110)^a^	66.7±4.4^a^
	Stressed	172	87.6±4.4 (155)^a^	41.7±8.4 (68)^b^	39.4±3.9^b^

a–b: In the same column, values without a common letter in their superscripts differ (P<0.05) within oocyte treatments.

### Effects of Maternal Predatory Stress During Follicular Growth/maturation on *in vivo* Embryo Development of Oocytes

To study the effect of maternal predatory stress during oocyte pre-maturation development on in vivo embryo development, mice were exposed to a cat for 24 h, starting 24 h after eCG injection (Fig. 1). At the end of the stress period, the mice were injected with hCG and placed with males overnight. Mice were examined for vaginal plugs the next morning, and females with vaginal plugs were sacrificed 3 days later for embryo collection. Whereas effects of maternal stress on pre-implantation development were examined by recording numbers of blastocysts obtained per donor mouse, the effects on post-implantation development were examined by observing the number and birth weight of young per recipient after embryo transfer. On each experimental day, whereas the number of blastocysts collected from each donor mouse was recorded in all the females that had shown vaginal plugs, embryo transfer and cell counting were conducted only when the number of blastocysts obtained was adequate for cell counting (5 per donor mouse) and for transfer to recipients (15 per recipient). Results showed that the number of blastocysts obtained per donor mouse was significantly lower in the stressed than in the unstressed mice (Table 3). Following embryo transfer, the average number of young per recipient from stressed donors was significantly lower than from controls, but average birth weight of the young was significantly higher than offspring of unstressed donors. Because cell counts per blastocyst did not differ between stressed and unstressed mice (Table 3), the decreased number of young per recipient in stressed donors suggested detrimental effects of maternal predatory stress on oocyte post-implantation development. Together, the results suggest that maternal predatory stress during the oocyte pre-maturation stage reduces oocyte developmental potential at both pre- and post-implantation stages.

**Table 3:** In vivo embryo development of oocytes after mice were exposed to cat for 24 h starting from 24 h after eCG injection.

Treatment	Donor mice	Blastocysts obtainedper donor mouse	Cell numberper blastocyst	Recipientmice	Average number ofyoung per recipient	Birth weight of young (g)
Control	17	20.2±3.3^a^	49.0±1.2^a^	15	8.6±0.5^a^	1.89±0.04^a^
Stressed	18	7.9±2.0^b^	48.3±1.5^a^	12	3.2±0.4^b^	2.08±0.06^b^

a–b: Values without a common letter in their superscripts differ significantly (P<0.05) within columns.

## Discussion

Studies have shown that exposure of rats and mice to natural predators or to their odors induces anxiety-like states [23]–[27] and stress hormone elevations [32]–[34]. Both anxiety-like behaviors and plasma cortisol concentrations were therefore measured in the present study to evaluate mouse responses to our predatory stress system. Results indicated that levels of anxiety and plasma cortisol each were significantly higher in stressed mice than in unstressed controls at measured times during 24-h exposure to cats. This verified that our predatory stress system with the test cat renewed at 6-h intervals was effective across time in inducing mouse stress responses. Results also revealed a positive correlation between cortisol concentrations and scores of anxiety-like behavior during cat exposure. Studies in human beings have also demonstrated a significant direct correlation between cortisol and anxiety [39], [40].

This study additionally showed that, although plasma cortisol in the stressed mice 1-h after termination to cat exposure declined to below that of control mice, their anxiety scores remained higher than those of the controls. Previous studies also have reported carryover increases in anxiety-like behavior after a brief exposure of rats or mice to a cat [25], [41]. Thus, researchers have suggested that the anxiety increasing after-effect of predator stress in rodents be used to study the mechanism for the posttraumatic stress disorder (PTSD) in humans, because PTSD patients also showed low urinary free-cortisol levels while still displaying overt signs of anxiety and depression [42].

Although corticosterone is considered to be the main glucocorticoid involved in regulation of stress responses in rodents, several studies have reported cortisol increase in plasma and adrenal glands following stress of mice [43]–[46]; and many studies have used cortisol as the index for stress activation in mice [19], [47]–[49] and rats [50]–[52]. Both the present results and our previous study [19] indicate that cortisol concentrations in unstressed control mice were relatively lower in blood samples collected early in the morning than in those collected in the afternoon. Serum corticosterone concentrations are also reported to be significantly lower in the morning than in the afternoon in both mice [53] and rats [54]. The present data and our previous study [19] showed that serum cortisol levels were markedly increased in response to both restraint and predatory stress. Similarly, significant elevation of corticosterone has been reported in both mice [53] and rats [55], [56] after restraint or noise stress. Furthermore, preliminary observations from our laboratory (unpublished) suggest that serum levels of both cortisol and corticosterone were elevated significantly following restraint stress of mice during the second 24-h after eCG injection and that cortisol (from 13 to 54 ng/ml) was raised to a much greater extent than corticosterone (from 250 to 310 ng/ml). Together, these data suggest that changes in corticosterone and cortisol concentrations in rodents are concordant under a variety of physiological conditions. In addition, it has been shown that cortisol exhibits much higher glucocorticoid potency than corticosterone [57]. We suggest therefore that cortisol concentration can be used as an indicator for stress activation in mice.

Oocyte maturation includes both nuclear maturation from the resumption of meiosis (GVBD) to the extrusion of the first polar body (metaphase II) and cytoplasmic maturation which is critical to fertilization and embryonic development. This study showed that although oocyte nuclear maturation and activation or fertilization were unaffected by maternal predatory stress, rates of blastocyst formation and cell count per blastocyst following either Sr^2+^ activation or in vitro fertilization were significantly lower in stressed mice than in non-stressed controls. To further confirm the effect of maternal stress on oocyte cytoplasmic maturation, pre- and post-implantation embryo development in vivo was observed by examining the number of blastocysts obtained per donor mouse and by outcomes from embryo transfer, respectively. Results showed that both the number of blastocysts obtained per donor mouse before embryo transfer and the average number of young per recipient after transfer of blastocysts with similar cell counts were significantly lower in the stressed than in the unstressed donor mice. Since our previous study [19] had shown that maternal restraint stress during the second 24-h after eCG had no marked effect on ovulation rate, it is suggested that the decrease in average blastocyst number in stressed donor mice is caused by impaired oocyte quality rather than by ovulation failure. Furthermore, our previous study indicated that whereas maternal restraint stress applied during the first 24-h period after eCG injection had no effect, that applied during the second 24-h significantly decreased oocyte blastocyst formation both in vitro and in vivo and also reduced the average number of young per recipient after embryo transfer, even though neither treatment affected oocyte nuclear maturation [19]. Taken together, our results suggest that maternal predatory stress applied at the pre-maturation stage of oocyte development significantly impairs ooplasmic maturation while having no effect on nuclear maturation.

Whereas in our previous study, birth weight decreased in offspring of stressed mice [19], it increased significantly in the present study. It should be noted that the difference between control and stressed embryos in the average number of young produced per recipient was greater (2.7 times, 8.6/3.2) in the present study than that (1.4 times, 11.6/8.3) in the previous study [19]. Perhaps implantation of a smaller number of embryos from stressed than unstressed donors provided a more accommodating uterine environment for implantation and rapid in utero growth. In other words, the higher birth weight after transfer of embryos from stressed donors may be an indirect effect of the decreased number of the young per recipient.

Early studies have suggested that nuclear maturation of oocytes is completed earlier and is less affected by environmental factors than cytoplasmic maturation. For example, with a similar nuclear maturation rate, whereas bovine oocytes harvested from dominant follicles immediately before the LH surge gave rise to a blastocyst rate of 50%, those originating from 2–6 mm follicles of slaughterhouse ovaries yielded a rate of about 30% following maturation and fertilization in vitro [36]. Although goat oocytes from both 2–3 mm and 3–4 mm follicles showed similar rates of nuclear maturation of about 80%, oocytes from 3–4 mm follicles achieved a blastocyst rate of about 30% as compared to those originating from 2–3 mm follicles yielding a rate of about 20% [58]. Furthermore, with a similar high nuclear maturation rate (over 90%), while mouse oocytes matured in TCM-199 produced a blastocyst rate of about 55%, only about 35% of those matured in modified α-MEM developed into blastocysts (unpublished data from this lab). Therefore, the observation that oocyte nuclear maturation was less affected by maternal psychological stress as compared with ooplasmic maturation may be due either to a low sensitivity of nuclear maturation to that stress or that the oocytes had already acquired competence to resume and complete meiosis by the time the stress was initiated. Taken together, the data suggest that the major events of cytoplasmic maturation of mouse oocytes take place during the second 24-h after eCG stimulation and that they are more vulnerable to stress than those of nuclear maturation.

In summary, although the predatory stress procedure is considered more psychological than the restraint procedure, it has rarely been used to study the effect of psychological stress on reproduction. Studies on the direct effect of psychological stress on oocytes are few, and the direct effect of maternal predatory stress on oocytes has not been reported. In the current study, we have successfully established a predatory stress system for mice with cats as predators and have studied for the first time the direct effect of predatory stress on oocyte developmental potential. Results showed that the predatory stress system we established with cats as predators effectively induced stress responses throughout the 24 h experimental period and that predatory stress applied at the oocyte pre-maturation stage resulted in significantly impaired oocyte developmental potential while having no impact on oocyte nuclear maturation, suggesting that cytoplasmic maturation of mouse oocytes was more vulnerable to stress than nuclear maturation. The present results, together with those from our previous study [19], have unequivocally shown that maternal psychological stress applied during follicular growth and maturation significantly diminishes the developmental potential of oocytes.

## Materials and Methods

### Ethics Statement

Mouse care and use were conducted exactly in accordance with the guidelines and approved by the Animal Research Committee of the Shandong Agricultural University, P. R. China (Permit number: 20010510). According to the guidelines of the committee, the animal handling staff (including each post-doc, doctoral or masters student) must be trained before using animals. Mice must be housed in a temperature-controlled room with proper darkness-light cycles, fed with a regular diet, and maintained under the care of the Experimental Animal Center, Shandong Agricultural University College of Animal Science and Vet Medicine. In the present study, mice were sacrificed by cervical dislocation. The only procedure performed on the dead animals was the collection of oocytes from the ovaries. A verbal consent was obtained from the owner of the cats for their use in this study.

Unless otherwise specified, all chemicals and reagents used were purchased from Sigma Chemical Co. (St. Louis, MO, USA).

### Animals

Mice of the Kunming breed were kept in a room with a constant temperature (22–25°C) and 14 h/10 h light-dark cycles, the dark starting at 8 PM. Sexually mature and gonadally intact male cats were used as predators. Female mice at the age of 6–8 weeks were injected with equine chorionic gonadotropin (eCG, 10 IU, ip) at 15∶30 pm followed 48 h later by human chorionic gonadotropin (hCG, 10 IU, ip). The hormone-stimulated mice were used for experiments at different times after eCG or hCG injection according the experimental design (Fig. 1). Both eCG and hCG used in this study were purchased from Ningbo Hormone Product Co., Ltd, P. R. China.

### Procedures for Predatory Stress

**Figure 3:**
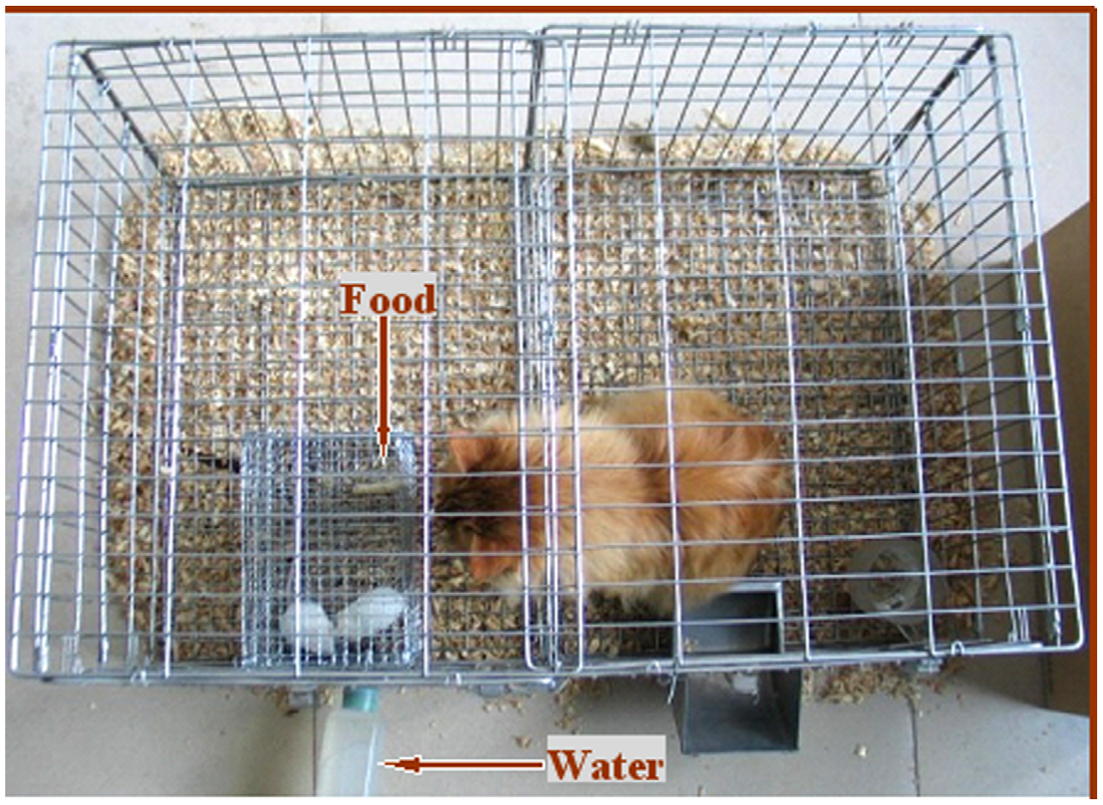
The cage-in-cage system used for the predator stress procedure, which consists of a large outer cage for the predator (cat) and a small inner cage for the prey (mouse). Both cages were made of steel-wire mesh; while the large cage measured 100 cm in length, 50 cm in width and 40 cm in height, the small cage measured 19 cm in length, 13 cm in width and 12 cm in height. The prey cage was fixed in such a position inside the predator cage that the cat and the mouse could see each other clearly from at least three sides.

Female mice were subjected to predatory stress for different times starting from 24 h (15∶30 pm the next day) following eCG injection (Fig. 1). A cage-in-cage system (Fig. 3) was built and used for the predatory stress procedure. The system consisted of a large outer cage for the predator (cat) and a small inner cage for the prey (mice). Both cages were made of steel-wire mesh; while the large cage measured 100 cm in length, 50 cm in width and 40 cm in height, the small cage measured 19 cm in length, 13 cm in width and 12 cm in height. The prey cage was fixed in such a position inside the predator cage that the cat and the mice could see each other clearly from at least three sides. Mice could move and take food and water freely while in their prey cage. For predatory stress, 2–3 mice were put in the prey cage, and one cat was placed in the predator cage. To ensure the continuation of stress, the predatory cats were deprived of food and water for half a day before use and the cat on duty was replaced every 6 h during the 24-h stress period. For controls, 2–3 mice were kept for the same periods in a prey cage that was placed in a separate room without cat exposure.

### Elevated Plus-maze Test (EPM)

The plus maze consisted of two open arms (30×6 cm), alternating in right angles with two closed arms (30×6×15 cm). The central platform delimited by the four arms was 36 cm^2^. The whole maze was elevated 50 cm above the floor. The animal's behavior on the maze was recorded via a video camera mounted on the ceiling above the center of the maze. The camera was connected to an Any-maze video tracking motion analysis system (Stoelting, Wood Dale, IL, USA) running on a personal computer. Before the start of the test, naïve mice were individually placed in a rectangular plastic arena for 5 min in order to habituate them to the test environment. To start the test, a mouse was placed in the central platform, facing an open arm, and was allowed to explore the maze for 5 min. Following a four-paw criterion, numbers of entries and time spent in each arm over the total exploration in both open and closed arms were calculated using the Any-maze software. The device was cleaned with 10% ethanol after each trial to effectively remove the scent of the previously tested animal.

### Hormone Assay

Mice were sacrificed by decollation, and trunk blood (about 1 ml) was collected into ice-cooled centrifugal tubes. Blood collection from stressed and control mice was always completed within 40 seconds after their release from the prey cage. Immediately after collection, the blood samples were centrifuged (1700×g, 10 min) at 4°C to separate serum. The serum collected was stored at −80°C until hormone assay. Serum cortisol levels were measured by radioimmunoassay at the Central Hospital of Tai-an City using commercial kits from Wei-Fang (3V) Bioengineering Co. Ltd., Wei-Fang city, P. R. China. The minimum level of detection of the cortisol assay was 1.0 ng/ml, and the intra- and inter-assay CVs were 5.3% and 6.7%, respectively.

### Oocyte Collection and Maturation *in vitro*

To recover immature oocytes at the germinal vesicle (GV) stage for in vitro maturation, the hormone-stimulated mice were sacrificed at 48 h after eCG administration. Large follicles (≥320 µm in diameter) on the ovary were ruptured in M2 medium to release cumulus-oocyte-complexes (COCs). Only COCs with more than three layers of unexpanded cumulus cells and containing oocytes >70 µm in diameter with a homogenous cytoplasm were selected for maturation. The recovered COCs were washed three times in M2 medium and once in maturation medium. The COCs were then cultured in groups of around 30 in 100-µl drops of maturation medium at 37.5°C in a humidified atmosphere of 5% CO_2_ in air. The maturation medium was TCM-199 (Gibco, Grand Island, New York, USA) supplemented with 10% (v/v) FCS (Gibco), 1 µg/ml 17 β-estradiol, 24.2 mg/L sodium pyruvate, 0.05 IU/ml FSH, 0.05 IU/ml LH and 10 ng/ml EGF.

### Oocyte Activation

The activating medium was Ca^2+^-free Chatot-Ziomek-Bavister (CZB) medium supplemented with 10 mM SrCl_2_. In vitro matured oocytes were recovered at 24 h of maturation culture and were stripped of their cumulus cells by pipetting in M2 containing 0.1% hyaluronidase. After being washed twice in M2 and once in the activating medium, the oocytes were incubated first in activating medium for 2.5 h and then in regular CZB without SrCl_2_ for 3.5 h at 37.5°C in a humidified atmosphere with 5% CO_2_ in air. Both the activating medium and CZB for subsequent short culture of oocytes were supplemented with 5 µg/ml cytochalasin B to diploidize the parthenotes. Six hours after the onset of activation treatment, the oocytes were examined with a microscope for the evidence of activation. Oocytes were considered activated when each contained one or two well developed pronuclei.

### Fertilization *in vitro*

Masses of dense sperm were collected from the cauda epididymis of fertile male mice and were placed at the bottom of a test tube containing T6 medium supplemented with 10 mg/ml BSA. After 3–5 min, the supernatant containing highly motile spermatozoa was removed and capacitated in the same medium under mineral oil at 37°C for 1.5 h. In vitro matured oocytes were collected after 14 h of maturation culture, and after being washed in the fertilization medium (T6 containing 20 mg/ml BSA), the oocytes were placed in fertilization drops (20 oocytes per 40-µl drop). Capacitated sperm were added to the fertilization drops to give a final sperm concentration of about 1×10^6^/ml. After 6 h of incubation, oocytes were observed under a microscope for fertilization. Oocytes with two pronuclei and the second polar body were selected and cultured for development.

### Embryo Culture

The Sr^2+^-activated oocytes and IVF zygotes were cultured for 4 d in the regular CZB without CB (20 embryos per 60-µl drop) at 37.5°C under humidified atmosphere with 5% CO_2_ in air. Glucose (5.5 mM) was added to CZB when embryos were cultured beyond the 3- or 4-cell stages. At the end of culture, embryo development was examined and some of the blastocysts were stained with Hoechst 33342 for cell number counting.

### Collection and Transfer of *in vivo* Embryos

Both control and stressed mice used for embryo donors were injected with hCG at 48 h after eCG injection. Immediately following hCG injection, the mice were placed with the males overnight. Mice were examined for presence of a vaginal plug the next morning (day 0 p.c.). In the afternoon of day 3 p.c., female mice having a vaginal plug were sacrificed and their uteri were flushed for embryos. After the numbers of blastocysts obtained per donor mouse were recorded, five blastocysts from each mouse were randomly selected for counting blastocyst cells and the rest were used for embryo transfer.

For embryo transfer, pseudo-pregnant recipients were prepared as reported previously [59]. The 3.5-day p.c. embryos were transferred into the uterine horns of the 2.5-day p.c. pseudo-pregnant recipients. Fifteen embryos were transferred to each recipient, 8 or 7 embryos per uterine horn.

### Data Analysis

There were at least three replicates for each treatment unless otherwise stated. Data were analyzed using SPSS 11.5 (Statistics Package for Social Sciences, Chicago, IL). Data were compared using one-way ANOVA followed by least significant difference (Duncan) post hoc tests. The percentage data were arc sine transformed, and assumptions that the transformed data were normal (Shapiro-Wilk and Kolmogorov-Smirnov tests for normality) and population variances were all equal (Levene test) were checked prior to performing the ANOVA. Data are expressed as mean ± SE and differences were considered significant at P<0.05.
